# Synthesis, crystal structure, and Hirshfeld surface analysis of 3-ferrocenyl-1-(pyridin-2-yl)-1*H*-pyrazol-5-amine

**DOI:** 10.1107/S2056989023008101

**Published:** 2023-09-19

**Authors:** Delara Joekar, Lana K. Hiscock, Louise N. Dawe

**Affiliations:** a Department of Chemistry and Biochemistry, Wilfrid Laurier University, 75 University Ave. W., Waterloo, Ontario, N2L 3C5, Canada; Vienna University of Technology, Austria

**Keywords:** crystal structure, amino­pyrazole, ferrocene, Hirshfeld surface analysis

## Abstract

A key step towards utilizing polynuclear metal-based systems in magnetic device applications involves the careful design of ligands. This strategic planning aims to produce metal assemblies that exhibit some kind of ‘switch’ mechanism. Towards this end, a ligand that incorporates a redox-active functional group (ferrocene) is reported. This communication presents the multi-step synthesis, characterization (^1^H and ^13^C NMR), and structural analysis (single-crystal X-ray diffraction and Hirshfeld surface analysis) of 3-ferrocenyl-1-(pyridin-2-yl)-1*H*-pyrazol-5-amine, [Fe(C_5_H_5_)(C_13_H_11_N_4_)]. Supra­molecular features, including π–π stacking and hydrogen bonding are qu­anti­fied, while a database search reveals the unique combination of mol­ecular moieties, which offer future opportunities for studies to involve simultaneous Lewis acid and base coordin­ation.

## Abstract 2

The multi-step synthesis, characterization, and structural examination (single-crystal X-ray diffraction and Hirshfeld surface analysis) of 3-ferrocenyl-1-(pyridin-2-yl)-1*H*-pyrazol-5-amine (C_18_H_16_FeN_4_), are reported. The supra­molecular characteristics, including π–π stacking and hydrogen bonding, are discussed, and a database exploration highlights the distinctive combination of mol­ecular components.

## 1. Chemical context

We have previously reported a pyrazole-based ligand scaffold, which incorporates groups for both cation and anion coordin­ation, as well as the opportunity for functionalization with other moieties for practical applications, for example, fluorescent tags (Hiscock *et al.*, 2019 ▸), or in the area of mol­ecular magnetism. 
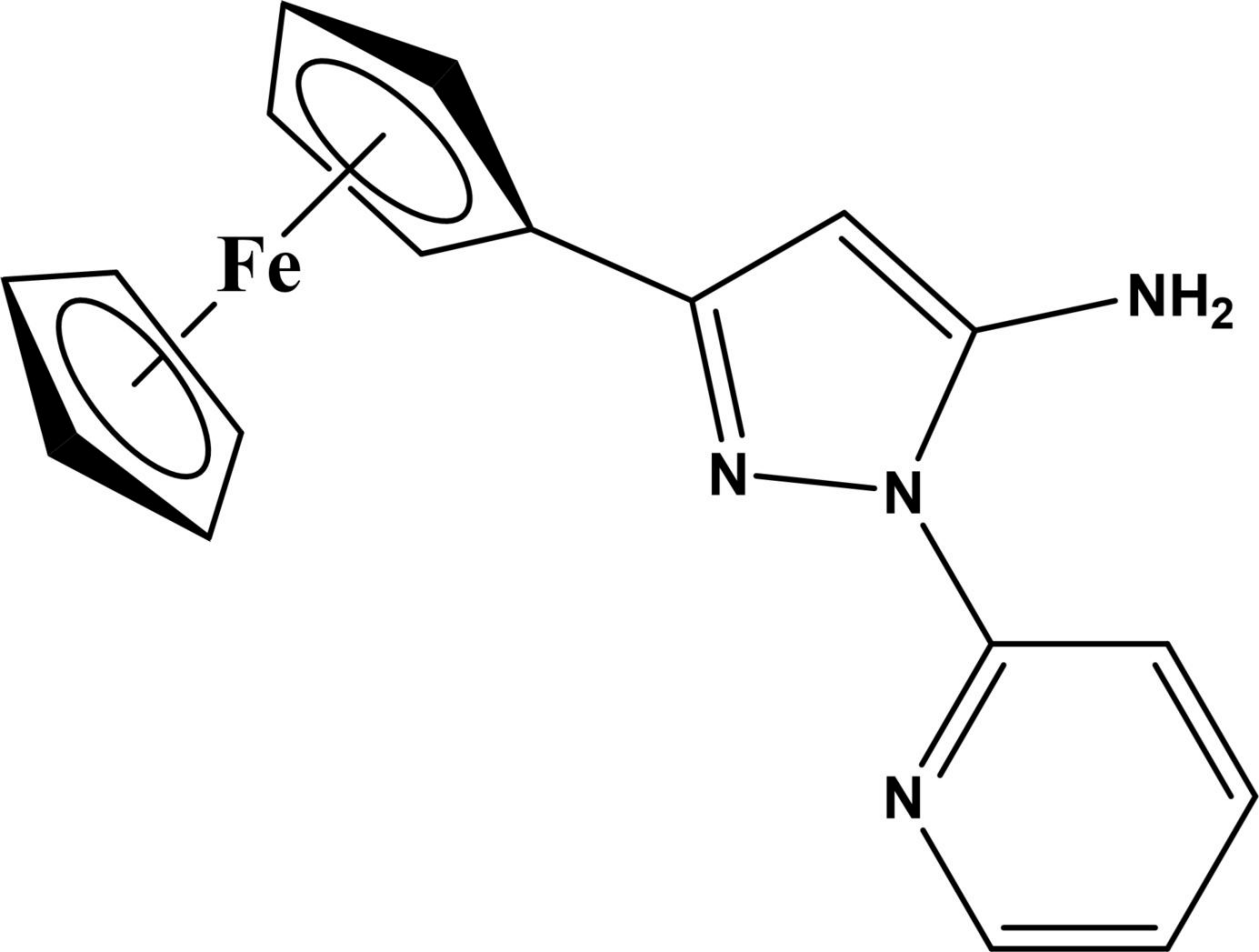


One step towards achieving magnetic device applications for polynuclear metal-based systems is the strategic design of ligands such that resulting metal assemblies possess some type of ‘switch’ [electrochemical, photo-induced, or other (Cador *et al.*, 2019 ▸)]. As an example, a single ion magnet switching process with a bis-di­amino­ferrocene-based ligand for Dy^III^ yielded a chemically (iodine) induced one-electron reduction (Dickie *et al.*, 2017 ▸). In this reversible process, a change in magnetization dynamics (in the absence of an applied DC field) characterized this system as an ‘on/off’ switch for slow magnetic relaxation.

Herein, the synthesis, characterization, and structural features of 3-ferrocenyl-1-(pyridin-2-yl)-1*H*-pyrazol-5-amine (**1**) are described. This ligand design enables future opportunities, as the substituent on the unfunctionalized pyrazole carbon atom can be varied to tune the metal coordination environment, for which single ion magnets are sensitive (Marin *et al.*, 2021 ▸; Gálico *et al.*, 2019 ▸).

## 2. Structural commentary

**Figure 1:**
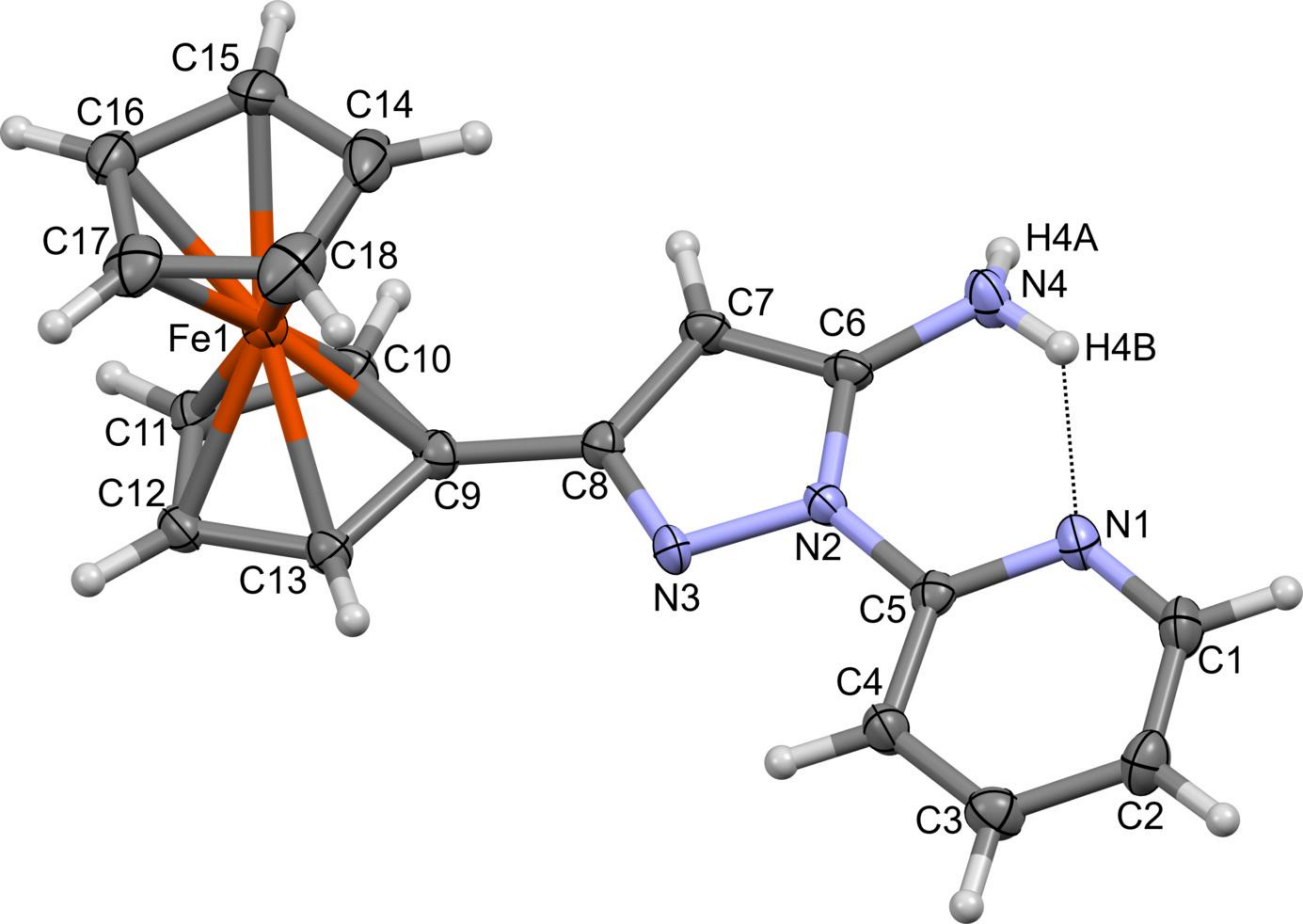
The asymmetric unit of **1**, shown with displacement ellipsoids at the 50% probability level and hydrogen atoms as fixed-size spheres with radius of 0.15 Å. The intra­molecular hydrogen bond is represented as a dashed line.

The mol­ecular structure of **1** is shown in Fig. 1 ▸. A Mogul geometry search (Cottrell *et al.*, 2012 ▸; Bruno *et al.*, 2004 ▸) revealed only one unusual bond angle, present in the pyrazole ring, formed by C7—C8—N3. The experimental value reported for this angle in **1** is 112.3 (2)°, while the Mogul search revealed a mean value of 111.28° with a standard deviation of 0.48° based on 33 observations in the Cambridge Structural Database (Groom *et al.*, 2016 ▸). It is noted that despite being flagged as unusual, the value for **1** lies within three standard deviations of that reported from the Mogul search.

In **1**, an intra­molecular hydrogen bond [graph-set notation 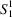 (6)] from the pyrazole amine group to the pyridyl nitro­gen acceptor (N4—H4*B*⋯N1; Fig. 1 ▸, Table 1 ▸) facilitates a near planar orientation of the pyridyl (py) and pyrazole (pz) rings [dihedral py–pz twist angle of 3.16 (3)°]. The orientation of the ferrocenyl cyclo­penta­dienyl ring (cp; C9–13) that is directly bound to the pyrazole ring exhibits a greater twist from planarity, with an observed cp–pz dihedral angle of 12.28 (12)°. The ferrocenyl cyclo­penta­dienyl rings in **1** are approximately eclipsed, with a dihedral angle of 3.8 (4)°.

## 3. Supra­molecular features and Hirshfeld surface analysis

**Figure 2:**
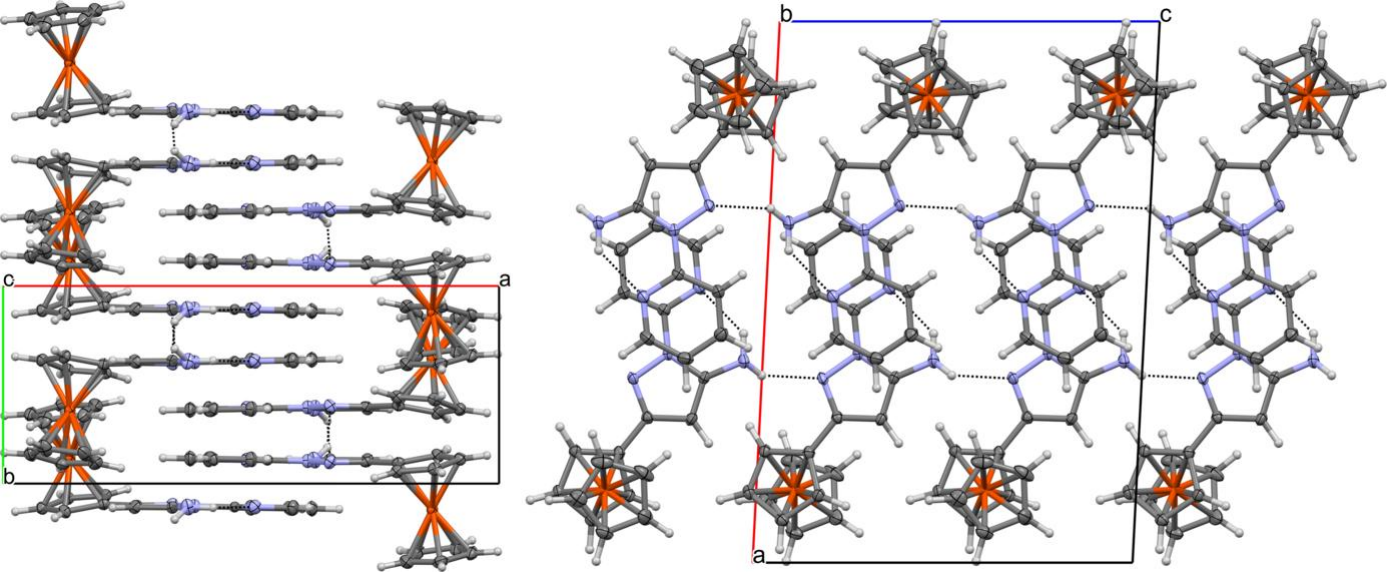
Packing diagrams for **1**, represented with displacement ellipsoids at the 50% probability level; (left) viewed down the *c* axis to show short contacts between pz–py planes of adjacent mol­ecules; (right) viewed down the *b* axis to show inter­molecular hydrogen-bonding perpendicular to the pz–py inter­planar inter­actions.

Examination of the crystal packing for **1** reveals short contacts between the mean pz–py planes, parallel to the *b* axis, with plane-to-plane centroid separations (*i.e.* shortest distance between planes) of 3.4790 (18) Å and a plane-to-plane shift of 2.006 (3) Å [measured from mol­ecules generated by symmetry operations (ii) *x*, *y*, *z* to (iii) 1 − *x*, *y* − 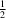 , 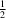 − *z*; Fig. 2 ▸]. Inter­molecular hydrogen bonding from the pyrazole amine group to an adjacent pyrazole nitro­gen acceptor (N4—H4*A*⋯N3^i^; Table 1 ▸) yields infinite chains [graph-set notation 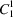 (5)] parallel to the *c* axis.

**Figure 3:**
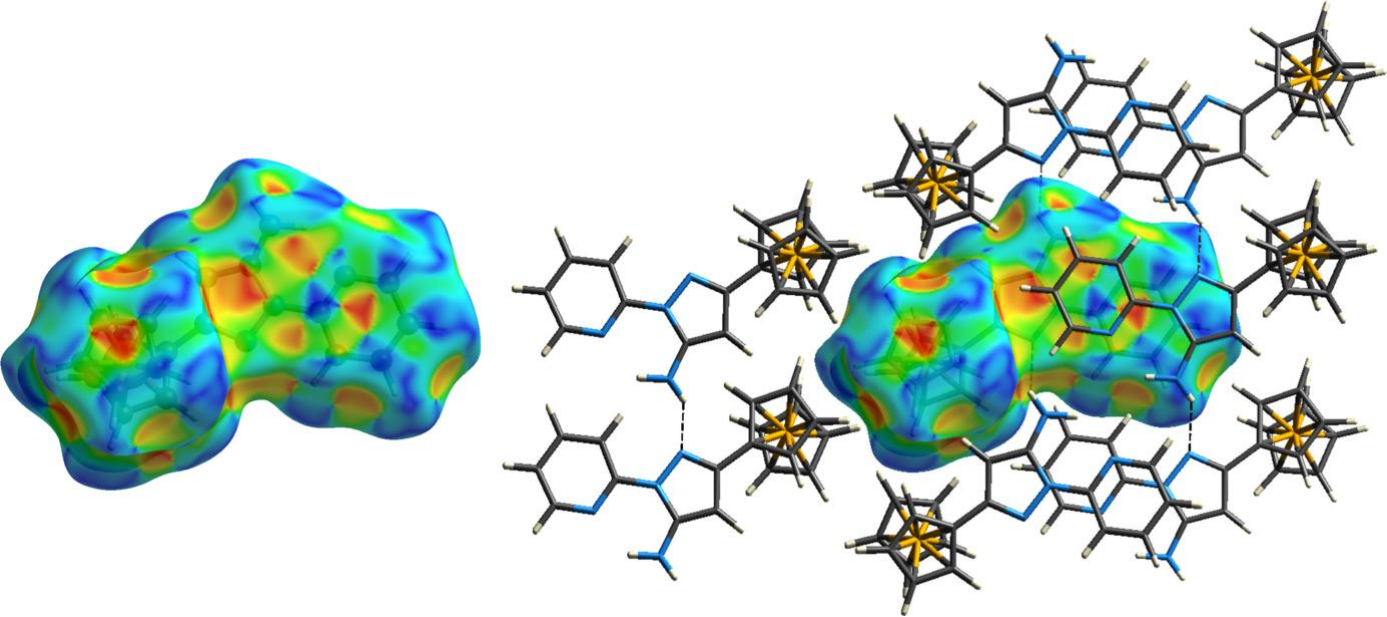
(Left) Hirshfeld shape index surface for the asymmetric unit of **1**, viewed down the *b* axis; and (right) with symmetry-related mol­ecules making short contacts with the asymmetric unit. Transparent surface representations, with ball-and-stick mol­ecular model on the left, and with mol­ecular bonds represented as tubes and hydrogen bonds as dashed lines on the right. Hydrogen atoms were generated in normalized neutron *X*—H positions by *CrystalExplorer17* (Spackman *et al.*, 2021 ▸).

**Figure 4:**
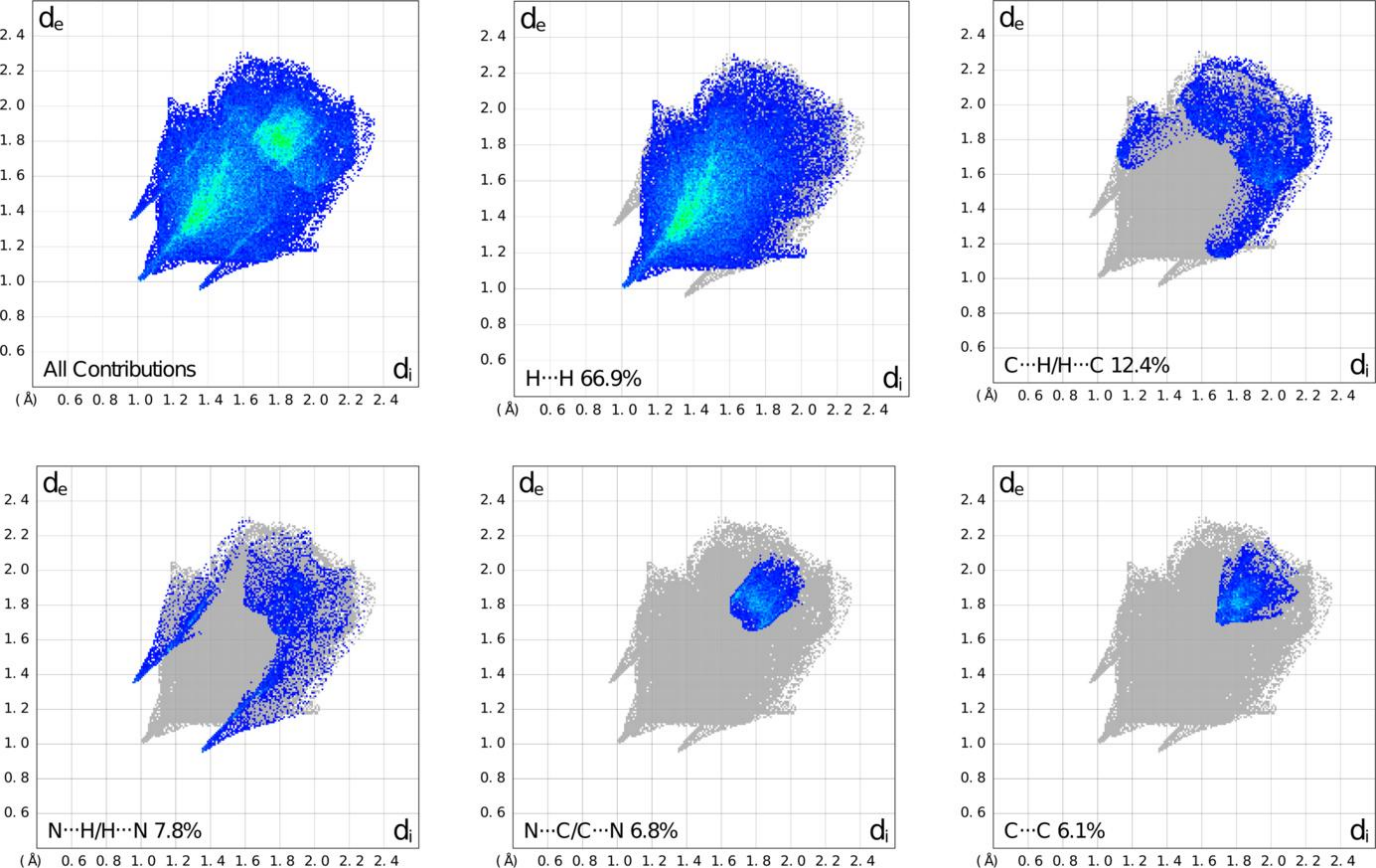
Fingerprint plots showing all close contacts in the crystal structure of **1** (top left), and (other plots) the contributions of the total inter­actions by H⋯H, C⋯H, N⋯H, N⋯C and C⋯C contacts. Plots were generated using *CrystalExplorer17* (Spackman *et al.*, 2021 ▸).

Hirshfeld surface analysis (Spackman & Jayatilaka, 2009 ▸) was performed using *CrystalExplorer17* (Spackman *et al.*, 2021 ▸). Examination of the shape-index plot (Fig. 3 ▸) shows the same short pz–py planar contacts and perpendicular N—H⋯N hydrogen-bonding inter­actions, but also additional short contacts, indicated as red hollows (shape-index <1) and blue bumps (shape-index >1) representing complementary inter­molecular inter­action between donors and acceptor groups, respectively (Tan *et al.*, 2019 ▸). These inter­actions are qu­anti­tatively summarized as 2D fingerprint plots (Fig. 4 ▸). In these plots, *d*_i_ is plotted on the *x*-axis and represents the distance to the nearest nucleus inside the Hirshfeld surface, and *d*_e_ is plotted on the *y*-axis, and represents the distance from the Hirshfeld surface to the nearest nucleus outside the surface. These fingerprint plots indicate weak (blue and blue–green) van der Waals H⋯H contacts as the dominant packing inter­action (66.9% of the overall surface) in **1**, with C⋯H/H⋯C contacts [*i.e.* C—H⋯π/π⋯C—H contacts (Tan *et al.*, 2019 ▸)] contributing 12.4% of the Hirshfeld area, and N⋯H/H⋯N, N⋯C/C⋯N, and C⋯C inter­actions contributing 7.8%, 6.8% and 6.1% of inter­actions, respectively. Note that, as expected, these plots are pseudo-mirrored along the diagonal, *i.e.* where *d*_e_ and *d*_i_ have the same value.

## 4. Database survey and conclusion

A search of the Cambridge Structural Database (Conquest Version 2023.1.0; CSD version 5.44 with April 2023 updates; Groom *et al.*, 2016 ▸) yielded 6635 carbon-functionalized mono-substituted ferrocene structures. Narrowing the search parameters to monosubstituted 3-ferrocenyl-1*H*-pyrazole structures yielded 96 structures with available coordinates, while further limiting the search to require the presence of a 1*H*-pyrazol-5-amine group resulted in zero previously reported structures. This demonstrates the unique combination of elements in the mol­ecular structure, each of which have been incorporated for a purpose (redox activity, cation coordin­ation, and hydrogen bonding), which we hope to demonstrate in future studies.

## 5. Synthesis and crystallization

^1^H NMR and ^13^C NMR spectra were recorded on an Agilent Technologies Varian Unity Inova 300 or 400 MHz NMR spectrometer using the indicated deuterated solvents purchased from Sigma-Aldrich. Chemical shifts are reported in δ scale in p.p.m. using the residual solvent peak (CDCl_3_, δ = 7.260) as reference. 2-Hydrazinyl­pyridine was prepared from 2-bromo­pyridine using a modified literature procedure (Klingele *et al.*, 2010 ▸), as we have previously reported (Hiscock *et al.*, 2019 ▸). α-Chloro­acetyl­ferrocene (Yang *et al.*, 2007 ▸) and 3-oxo-3-(ferrocen­yl)propane­nitrile (Rao & Muthanna, 2016 ▸) were also prepared *via* modified literature procedures. All other reagents and starting materials were purchased from Sigma-Aldrich and used as purchased. Melting points were determined on a Mel-Temp Electrothermal melting point apparatus and are uncorrected.

**Figure 5:**
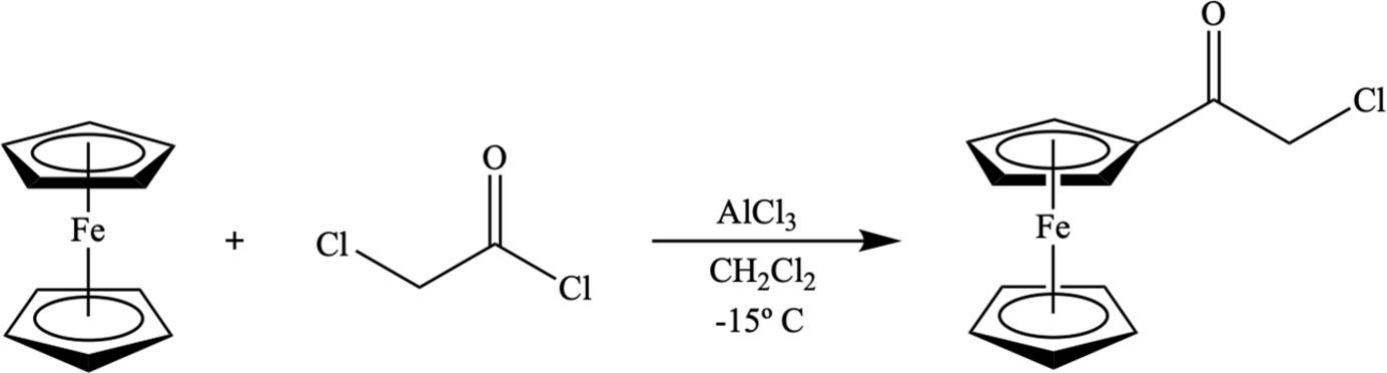
Schematic synthesis of α-chloro­acetyl­ferrocene.

Synthesis of α-chloro­acetyl­ferrocene is schematically shown in Fig. 5 ▸. Dry CH_2_Cl_2_ (50 ml) was placed in an oven-dried 250 ml round-bottom flask equipped with a stir bar under a nitro­gen atmosphere. Purified ferrocene (2.00 g, 11.0 mmol, 1.5 eq) was added to the flask producing a clear orange mixture. 2-Chloroacetyl chloride (0.60 mL, 7.2 mmol, 1.0 eq) was also added to the mixture. The round-bottom flask was then placed in an ice bath (NaCl/ice, 1:3). AlCl_3_ (1.43 g, 7.20 mmol, 1.0 eq) was gradually added to the mixture in three equal portions (0.47 g) every 15 min, resulting in a cloudy dark-purple mixture. It was then stirred at room temperature under a nitro­gen atmosphere for 24 h. Distilled water (50 ml) was added to the flask while stirring in an ice bath. The cloudy dark-purple mixture was placed in a separatory funnel and washed with distilled water (2 × 25 ml) and saturated NaHCO_3_ (2 × 25 ml). The organic layer was dried over MgSO_4_ and the volume was reduced by rotary evaporation. The dark-brown solid was purified by column chromatography (SiO_2_, 5% EtOAc/PhMe). Rotary evaporation of the fraction containing the product gave α-chloro­acetyl­ferrocene, a dark-red powdery solid (0.260 g, 1.00 mmol, 9%). ^1^H NMR (300 MHz, CDCl_3_) δ: 4.81 (*s*, 2H), 4.63 (*s*, 2H), 4.27 (*s*, 5H), 3.76 (*s*, 2H); ^13^C NMR (75 MHz, CDCl_3_) δ: 114.2, 76.4, 73.6, 70.6, 69.8, 29.7.

**Figure 6:**
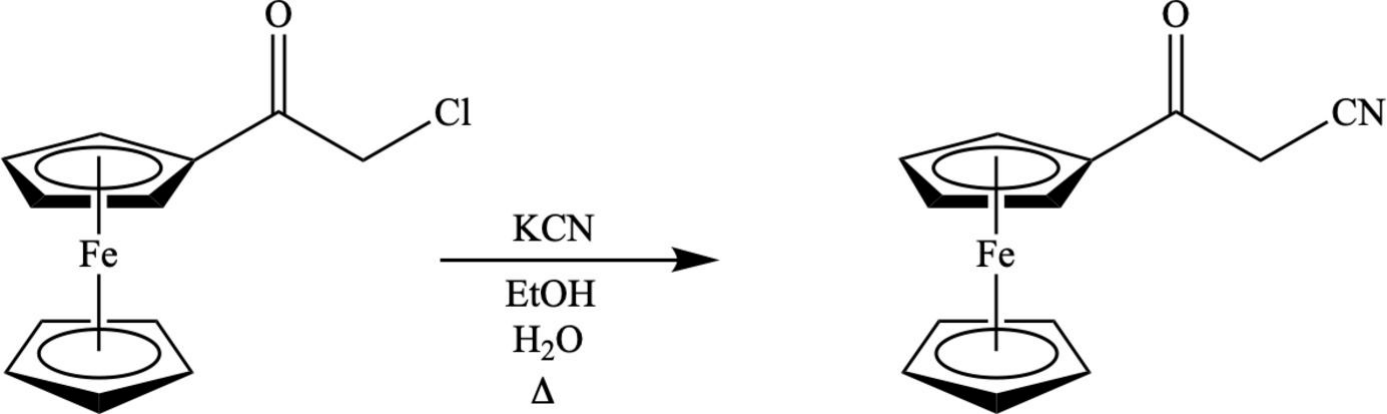
Schematic synthesis of 3-oxo-3-(ferrocen­yl)propane­nitrile.

Synthesis of 3-oxo-3-(ferrocen­yl)propane­nitrile is schematically shown in Fig. 6 ▸. KCN (0.733 g, 11.0 mmol, 2.0 eq) was placed in a 100 ml round-bottom flask equipped with a stir bar. Distilled water (6.0 ml) and ethanol (17 ml) were added followed by α-chloro­acetyl­ferrocene (1.00 g, 5.10 mmol, 1.0 eq), which resulted in a clear dark-red mixture. It was refluxed for 48 h. The following step was performed with great care: In a very well-ventilated fumehood, HCl (12 *M*, 1.0 ml) was added and nitro­gen was bubbled through the solution for 1 h. The volume was reduced by rotary evaporation (10 ml NaOH in the trap) yielding a brown powder. It was dissolved in di­chloro­methane (30 ml) and washed with distilled water (3 × 25 ml), K_2_CO_3_ (3 × 25 ml), and brine (1 × 25 ml). The organic layer was dried over MgSO_4_ and the volume was reduced by rotary evaporation to give 3-oxo-3-(ferrocen­yl)propane­nitrile, a dark-brown powdery solid (0.185 g, 0.964 mmol, 19%). ^1^H NMR (300 MHz, CDCl_3_) δ: 4.81 (*s*, 2H), 4.63 (*s*, 2H), 4.27 (*s*, 5H), 3.76 (*s*, 2H); ^13^C NMR (75 MHz, CDCl_3_) δ: 190.7, 114.2, 76.4, 73.6, 70.6, 69.8, 29.7.

**Figure 7:**
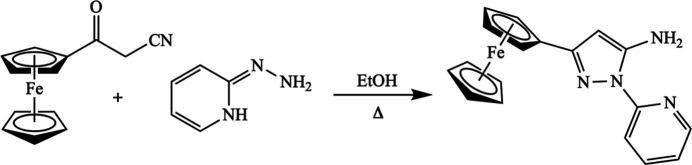
Schematic synthesis of 3-ferrocenyl-1-(pyridin-2-yl)-1*H*-pyrazol-5-amine.

Synthesis of 3-ferrocenyl-1-(pyridin-2-yl)-1*H*-pyrazol-5-amine is schematically shown in Fig. 7 ▸. 2-Hydrazinyl­pyridine (2.80 g, 25.8 mmol, 1.0 eq) and 3-oxo-3-(ferrocen­yl)propane­nitrile (5.04 g, 25.8 mmol, 1.0 eq) were placed in ethanol (20 ml) in a 100 ml round-bottom flask equipped with a condenser and a stir bar. The resulting dark-brown mixture was refluxed for 48 h. The volume was then reduced by rotary evaporation. The product was purified by column chromatography (SiO_2_, 5% EtOAc/PhMe). Rotary evaporation gave 3-ferrocenyl-1-(pyridin-2-yl)-1*H*-pyrazol-5-amine, a brown–orange crystalline solid containing X-ray quality single-crystals (0.137 g, 0.400 mmol, 2%). ^1^H NMR (400 MHz, CDCl_3_) δ: 8.37–8.35 (*ddd*, *J* = 5.0, 1.95, 0.85 Hz, 1H), 8.08–8.05 (*dt*, *J* = 8.49, 0.96 Hz, 1H), 7.86–7.80 (*d*, *J* = 0.75 Hz, 1H), 7.13–7.09 (*ddd*, *J* = 7.38, 4.96, 1.06 Hz, 1H), 5.98 (*s*, 2H), 5.66 (*s*, *J* = 0.64 H, 1H), 4.73 (*s*, 2H), 4.32 (*s*, 2H), 4.15 (s, 5H); ^13^C NMR (100 MHz, CDCl_3_) δ: 154.9, 152.3, 149.7, 146.6, 138.8, 119.7, 113.9, 87.3, 78.2, 69.4, 68.6, 66.8.

## 6. Refinement

Crystal data, data collection and structure refinement details are summarized in Table 2 ▸. All the hydrogen atoms, except H4*A* and H4*B*, were positioned geometrically (C—H = 0.95 Å) and refined using a riding model with *U*_iso_(H) =1.2*U*_eq_ of the carrier atom. Amine hydrogen atoms, H4*A* and H4*B*, were introduced in their difference electron density map positions and refined isotropically.

## Supplementary Material

Crystal structure: contains datablock(s) I. DOI: 10.1107/S2056989023008101/wm5693sup1.cif

Structure factors: contains datablock(s) I. DOI: 10.1107/S2056989023008101/wm5693Isup3.hkl

CCDC reference: 2287368

Additional supporting information:  crystallographic information; 3D view; checkCIF report

## Figures and Tables

**Table 1:** Hydrogen-bond geometry (Å, °)

*D*—H⋯*A*	*D*—H	H⋯*A*	*D*⋯*A*	*D*—H⋯*A*
N4—H4*A*⋯N3^i^	0.86 (3)	2.44 (3)	3.210 (4)	150 (3)
N4—H4*B*⋯N1	0.88 (4)	2.05 (4)	2.749 (4)	136 (3)

Symmetry code: (i) 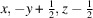 .

**Table 2:** Experimental details

Crystal data	
Chemical formula	[Fe(C_5_H_5_)(C_13_H_11_N_4_)]
*M*_r_	344.20
Crystal system, space group	Monoclinic, *P*2_1_/*c*
Temperature (K)	110
*a*, *b*, *c* (Å)	17.349 (8), 6.894 (3), 12.173 (5)
β (°)	92.878 (12)
*V* (Å^3^)	1454.0 (11)
*Z*	4
Radiation type	Mo *K*α
μ (mm^−1^)	1.04
Crystal size (mm)	0.15 × 0.10 × 0.04

Data collection	
Diffractometer	Bruker APEXII CCD
Absorption correction	Multi-scan (*SADABS*; Krause *et al.*, 2015 ▸)
*T*_min_, *T*_max_	0.829, 0.956
No. of measured, independent and observed [*I* > 2σ(*I*)] reflections	37974, 2578, 1877
*R*_int_	0.105
(sin θ/λ)_max_ (Å^−1^)	0.596

Refinement	
*R*[*F* ^2^ > 2σ(*F* ^2^)], *wR*(*F* ^2^), *S*	0.036, 0.078, 1.02
No. of reflections	2578
No. of parameters	216
H-atom treatment	H atoms treated by a mixture of independent and constrained refinement
Δρ_max_, Δρ_min_ (e Å^−3^)	0.42, −0.32

Computer programs: *APEX2* (Bruker, 2012 ▸), *SAINT* (Bruker, 2018 ▸), SHELXT (Sheldrick, 2015*a* ▸), *SHELXL* (Sheldrick, 2015*b* ▸), and *OLEX2* (Dolomanov *et al.*, 2009 ▸).

## supplementary crystallographic information

### Crystal data

**Table:**

[Fe(C_5_H_5_)(C_13_H_11_N_4_)]	*F*(000) = 712
*M**_r_* = 344.20	*D*_x_ = 1.572 Mg m^−^^3^
Monoclinic, *P*2_1_/*c*	Mo *K*α radiation, λ = 0.71073 Å
*a* = 17.349 (8) Å	Cell parameters from 7455 reflections
*b* = 6.894 (3) Å	θ = 3.4–24.7°
*c* = 12.173 (5) Å	µ = 1.04 mm^−^^1^
β = 92.878 (12)°	*T* = 110 K
*V* = 1454.0 (11) Å^3^	Plate, orange
*Z* = 4	0.15 × 0.10 × 0.04 mm

### Data collection

**Table:**

Bruker APEXII CCD diffractometer	1877 reflections with *I* > 2σ(*I*)
φ and ω scans	*R*_int_ = 0.105
Absorption correction: multi-scan (*SADABS*; Krause *et al.*, 2015)	θ_max_ = 25.1°, θ_min_ = 2.4°
*T*_min_ = 0.829, *T*_max_ = 0.956	*h* = −20→20
37974 measured reflections	*k* = −8→8
2578 independent reflections	*l* = −14→14

### Refinement

**Table:**

Refinement on *F*^2^	Primary atom site location: dual
Least-squares matrix: full	Hydrogen site location: mixed
*R*[*F*^2^ > 2σ(*F*^2^)] = 0.036	H atoms treated by a mixture of independent and constrained refinement
*wR*(*F*^2^) = 0.078	*w* = 1/[σ^2^(*F*_o_^2^) + (0.0216*P*)^2^ + 2.158*P*] where *P* = (*F*_o_^2^ + 2*F*_c_^2^)/3
*S* = 1.01	(Δ/σ)_max_ < 0.001
2578 reflections	Δρ_max_ = 0.42 e Å^−^^3^
216 parameters	Δρ_min_ = −0.32 e Å^−^^3^
0 restraints	

### Special details

**Table:**

Geometry. All esds (except the esd in the dihedral angle between two l.s. planes) are estimated using the full covariance matrix. The cell esds are taken into account individually in the estimation of esds in distances, angles and torsion angles; correlations between esds in cell parameters are only used when they are defined by crystal symmetry. An approximate (isotropic) treatment of cell esds is used for estimating esds involving l.s. planes.

### Fractional atomic coordinates and isotropic or equivalent isotropic displacement parameters (Å^2^)

**Table:**

	*x*	*y*	*z*	*U*_iso_*/*U*_eq_	
Fe1	0.13466 (2)	0.63057 (6)	0.39894 (3)	0.01773 (13)	
N1	0.50809 (14)	0.3811 (4)	0.17719 (18)	0.0217 (6)	
N2	0.38601 (13)	0.3858 (4)	0.24937 (18)	0.0175 (5)	
N3	0.34008 (13)	0.3919 (4)	0.33927 (18)	0.0182 (5)	
N4	0.37249 (17)	0.3811 (4)	0.0502 (2)	0.0232 (6)	
H4A	0.3461 (18)	0.318 (5)	0.001 (3)	0.025 (9)*	
H4B	0.423 (2)	0.378 (6)	0.054 (3)	0.047 (11)*	
C1	0.58538 (17)	0.3775 (5)	0.1939 (2)	0.0249 (7)	
H1	0.615939	0.377992	0.131304	0.030*	
C2	0.62261 (17)	0.3733 (5)	0.2960 (2)	0.0266 (7)	
H2	0.677358	0.370090	0.303644	0.032*	
C3	0.57860 (17)	0.3738 (5)	0.3872 (2)	0.0248 (7)	
H3	0.602776	0.371625	0.458937	0.030*	
C4	0.49941 (16)	0.3774 (5)	0.3735 (2)	0.0220 (6)	
H4	0.467917	0.377177	0.435100	0.026*	
C5	0.46683 (16)	0.3813 (4)	0.2669 (2)	0.0162 (6)	
C6	0.34133 (16)	0.3791 (4)	0.1515 (2)	0.0173 (6)	
C7	0.26567 (16)	0.3809 (4)	0.1799 (2)	0.0195 (6)	
H7	0.220986	0.377176	0.131562	0.023*	
C8	0.26812 (16)	0.3896 (4)	0.2957 (2)	0.0169 (6)	
C9	0.20262 (16)	0.3951 (4)	0.3665 (2)	0.0184 (6)	
C10	0.12391 (16)	0.3580 (4)	0.3347 (2)	0.0205 (6)	
H10	0.104586	0.326560	0.262506	0.025*	
C11	0.07891 (17)	0.3757 (4)	0.4286 (2)	0.0206 (6)	
H11	0.024704	0.357569	0.430408	0.025*	
C12	0.12993 (17)	0.4254 (4)	0.5196 (2)	0.0201 (7)	
H12	0.115721	0.445806	0.593091	0.024*	
C13	0.20567 (17)	0.4393 (4)	0.4814 (2)	0.0191 (7)	
H13	0.250783	0.472386	0.524847	0.023*	
C14	0.16504 (19)	0.8494 (5)	0.2979 (3)	0.0344 (9)	
H14	0.199240	0.838866	0.239615	0.041*	
C15	0.08432 (19)	0.8210 (5)	0.2885 (3)	0.0313 (8)	
H15	0.054821	0.787232	0.223430	0.038*	
C16	0.05549 (18)	0.8521 (5)	0.3936 (3)	0.0272 (7)	
H16	0.002936	0.844053	0.411569	0.033*	
C17	0.11837 (18)	0.8970 (4)	0.4671 (3)	0.0293 (8)	
H17	0.115362	0.923602	0.543339	0.035*	
C18	0.18652 (19)	0.8960 (5)	0.4084 (3)	0.0347 (8)	
H18	0.237283	0.921796	0.437669	0.042*	

### Atomic displacement parameters (Å^2^)

**Table:**

	*U*^11^	*U*^22^	*U*^33^	*U*^12^	*U*^13^	*U*^23^
Fe1	0.0171 (2)	0.0199 (2)	0.0163 (2)	0.0013 (2)	0.00189 (16)	0.0013 (2)
N1	0.0217 (14)	0.0254 (13)	0.0186 (13)	0.0004 (13)	0.0064 (11)	−0.0001 (13)
N2	0.0176 (13)	0.0225 (13)	0.0126 (11)	−0.0001 (12)	0.0015 (10)	0.0016 (12)
N3	0.0194 (13)	0.0231 (13)	0.0125 (12)	0.0038 (12)	0.0046 (10)	−0.0016 (11)
N4	0.0221 (16)	0.0333 (16)	0.0142 (13)	−0.0030 (14)	0.0029 (12)	−0.0051 (13)
C1	0.0211 (17)	0.0290 (17)	0.0254 (16)	−0.0003 (16)	0.0090 (13)	−0.0003 (16)
C2	0.0174 (16)	0.0306 (17)	0.0321 (18)	−0.0005 (16)	0.0043 (14)	−0.0026 (18)
C3	0.0237 (17)	0.0273 (16)	0.0229 (16)	−0.0036 (16)	−0.0017 (13)	0.0011 (16)
C4	0.0201 (16)	0.0288 (16)	0.0172 (15)	−0.0005 (16)	0.0027 (12)	0.0008 (16)
C5	0.0172 (15)	0.0133 (13)	0.0184 (14)	0.0009 (13)	0.0020 (12)	−0.0004 (14)
C6	0.0198 (16)	0.0181 (14)	0.0134 (14)	0.0009 (14)	−0.0027 (12)	−0.0009 (14)
C7	0.0203 (16)	0.0224 (15)	0.0153 (14)	0.0018 (15)	−0.0036 (12)	−0.0026 (14)
C8	0.0179 (15)	0.0167 (14)	0.0163 (14)	0.0014 (13)	0.0019 (12)	−0.0008 (13)
C9	0.0207 (16)	0.0178 (15)	0.0167 (15)	0.0007 (13)	0.0014 (12)	−0.0018 (13)
C10	0.0214 (16)	0.0225 (15)	0.0174 (15)	−0.0026 (15)	−0.0008 (12)	−0.0006 (14)
C11	0.0196 (16)	0.0214 (15)	0.0210 (15)	0.0004 (15)	0.0037 (12)	0.0023 (15)
C12	0.0239 (17)	0.0210 (16)	0.0155 (15)	0.0012 (13)	0.0037 (13)	0.0015 (12)
C13	0.0205 (17)	0.0206 (15)	0.0160 (15)	0.0016 (13)	−0.0001 (13)	−0.0013 (12)
C14	0.030 (2)	0.0301 (19)	0.045 (2)	0.0082 (16)	0.0199 (16)	0.0167 (18)
C15	0.0286 (19)	0.038 (2)	0.0273 (18)	0.0078 (16)	0.0037 (15)	0.0159 (15)
C16	0.0213 (17)	0.0259 (17)	0.0348 (18)	0.0042 (15)	0.0072 (14)	0.0088 (16)
C17	0.033 (2)	0.0200 (17)	0.0347 (18)	0.0048 (16)	0.0039 (16)	−0.0032 (15)
C18	0.0247 (18)	0.0235 (18)	0.056 (2)	−0.0034 (16)	0.0035 (17)	0.0041 (18)

### Geometric parameters (Å, º)

**Table:**

Fe1—C14	2.033 (3)	C4—C5	1.389 (4)
Fe1—C13	2.035 (3)	C4—H4	0.9500
Fe1—C18	2.040 (3)	C6—C7	1.374 (4)
Fe1—C10	2.040 (3)	C7—C8	1.409 (4)
Fe1—C17	2.041 (3)	C7—H7	0.9500
Fe1—C15	2.044 (3)	C8—C9	1.460 (4)
Fe1—C12	2.044 (3)	C9—C10	1.423 (4)
Fe1—C11	2.047 (3)	C9—C13	1.431 (4)
Fe1—C16	2.053 (3)	C10—C11	1.422 (4)
Fe1—C9	2.056 (3)	C10—H10	0.9500
N1—C5	1.335 (3)	C11—C12	1.425 (4)
N1—C1	1.346 (4)	C11—H11	0.9500
N2—N3	1.386 (3)	C12—C13	1.419 (4)
N2—C6	1.389 (3)	C12—H12	0.9500
N2—C5	1.408 (3)	C13—H13	0.9500
N3—C8	1.332 (3)	C14—C15	1.413 (5)
N4—C6	1.371 (3)	C14—C18	1.415 (5)
N4—H4A	0.86 (3)	C14—H14	0.9500
N4—H4B	0.88 (4)	C15—C16	1.413 (4)
C1—C2	1.372 (4)	C15—H15	0.9500
C1—H1	0.9500	C16—C17	1.410 (4)
C2—C3	1.378 (4)	C16—H16	0.9500
C2—H2	0.9500	C17—C18	1.412 (5)
C3—C4	1.376 (4)	C17—H17	0.9500
C3—H3	0.9500	C18—H18	0.9500
			
C14—Fe1—C13	127.63 (13)	N4—C6—N2	122.9 (3)
C14—Fe1—C18	40.67 (14)	C7—C6—N2	106.4 (2)
C13—Fe1—C18	107.40 (13)	C6—C7—C8	105.7 (2)
C14—Fe1—C10	118.24 (13)	C6—C7—H7	127.1
C13—Fe1—C10	68.51 (12)	C8—C7—H7	127.1
C18—Fe1—C10	151.59 (13)	N3—C8—C7	112.3 (2)
C14—Fe1—C17	67.89 (14)	N3—C8—C9	120.4 (2)
C13—Fe1—C17	118.39 (13)	C7—C8—C9	127.3 (3)
C18—Fe1—C17	40.47 (13)	C10—C9—C13	107.0 (2)
C10—Fe1—C17	166.61 (12)	C10—C9—C8	126.8 (2)
C14—Fe1—C15	40.56 (13)	C13—C9—C8	126.2 (3)
C13—Fe1—C15	165.73 (12)	C10—C9—Fe1	69.06 (17)
C18—Fe1—C15	68.51 (14)	C13—C9—Fe1	68.72 (16)
C10—Fe1—C15	108.17 (13)	C8—C9—Fe1	127.2 (2)
C17—Fe1—C15	68.03 (14)	C11—C10—C9	108.9 (2)
C14—Fe1—C12	165.82 (13)	C11—C10—Fe1	69.90 (17)
C13—Fe1—C12	40.72 (11)	C9—C10—Fe1	70.27 (17)
C18—Fe1—C12	127.85 (14)	C11—C10—H10	125.5
C10—Fe1—C12	68.40 (12)	C9—C10—H10	125.5
C17—Fe1—C12	108.61 (13)	Fe1—C10—H10	125.9
C15—Fe1—C12	152.40 (13)	C10—C11—C12	107.5 (3)
C14—Fe1—C11	152.03 (13)	C10—C11—Fe1	69.39 (17)
C13—Fe1—C11	68.67 (12)	C12—C11—Fe1	69.50 (17)
C18—Fe1—C11	166.21 (13)	C10—C11—H11	126.2
C10—Fe1—C11	40.71 (11)	C12—C11—H11	126.2
C17—Fe1—C11	128.65 (12)	Fe1—C11—H11	126.4
C15—Fe1—C11	118.53 (13)	C13—C12—C11	108.1 (3)
C12—Fe1—C11	40.76 (11)	C13—C12—Fe1	69.30 (16)
C14—Fe1—C16	67.80 (13)	C11—C12—Fe1	69.74 (17)
C13—Fe1—C16	152.29 (12)	C13—C12—H12	125.9
C18—Fe1—C16	68.10 (14)	C11—C12—H12	125.9
C10—Fe1—C16	128.67 (12)	Fe1—C12—H12	126.6
C17—Fe1—C16	40.30 (12)	C12—C13—C9	108.5 (3)
C15—Fe1—C16	40.36 (12)	C12—C13—Fe1	69.99 (17)
C12—Fe1—C16	119.12 (12)	C9—C13—Fe1	70.34 (17)
C11—Fe1—C16	108.84 (13)	C12—C13—H13	125.8
C14—Fe1—C9	107.51 (13)	C9—C13—H13	125.8
C13—Fe1—C9	40.94 (11)	Fe1—C13—H13	125.5
C18—Fe1—C9	117.62 (13)	C15—C14—C18	108.7 (3)
C10—Fe1—C9	40.67 (11)	C15—C14—Fe1	70.14 (18)
C17—Fe1—C9	151.77 (12)	C18—C14—Fe1	69.94 (19)
C15—Fe1—C9	127.65 (12)	C15—C14—H14	125.6
C12—Fe1—C9	68.66 (11)	C18—C14—H14	125.6
C11—Fe1—C9	68.71 (12)	Fe1—C14—H14	125.9
C16—Fe1—C9	166.01 (12)	C14—C15—C16	107.5 (3)
C5—N1—C1	116.6 (2)	C14—C15—Fe1	69.30 (18)
N3—N2—C6	111.1 (2)	C16—C15—Fe1	70.17 (18)
N3—N2—C5	119.3 (2)	C14—C15—H15	126.3
C6—N2—C5	129.6 (2)	C16—C15—H15	126.3
C8—N3—N2	104.5 (2)	Fe1—C15—H15	125.8
C6—N4—H4A	114 (2)	C17—C16—C15	108.1 (3)
C6—N4—H4B	113 (2)	C17—C16—Fe1	69.41 (18)
H4A—N4—H4B	122 (3)	C15—C16—Fe1	69.47 (18)
N1—C1—C2	123.9 (3)	C17—C16—H16	126.0
N1—C1—H1	118.1	C15—C16—H16	126.0
C2—C1—H1	118.1	Fe1—C16—H16	126.7
C1—C2—C3	118.3 (3)	C16—C17—C18	108.6 (3)
C1—C2—H2	120.8	C16—C17—Fe1	70.29 (18)
C3—C2—H2	120.8	C18—C17—Fe1	69.71 (19)
C4—C3—C2	119.5 (3)	C16—C17—H17	125.7
C4—C3—H3	120.2	C18—C17—H17	125.7
C2—C3—H3	120.2	Fe1—C17—H17	125.9
C3—C4—C5	118.1 (3)	C17—C18—C14	107.2 (3)
C3—C4—H4	121.0	C17—C18—Fe1	69.82 (19)
C5—C4—H4	121.0	C14—C18—Fe1	69.39 (19)
N1—C5—C4	123.6 (3)	C17—C18—H18	126.4
N1—C5—N2	116.6 (2)	C14—C18—H18	126.4
C4—C5—N2	119.8 (2)	Fe1—C18—H18	125.9
N4—C6—C7	130.6 (3)		
			
C6—N2—N3—C8	−0.2 (3)	Fe1—C9—C10—C11	−59.4 (2)
C5—N2—N3—C8	−178.3 (3)	C13—C9—C10—Fe1	58.5 (2)
C5—N1—C1—C2	0.3 (5)	C8—C9—C10—Fe1	−121.5 (3)
N1—C1—C2—C3	−0.4 (5)	C9—C10—C11—C12	0.4 (3)
C1—C2—C3—C4	0.4 (5)	Fe1—C10—C11—C12	−59.3 (2)
C2—C3—C4—C5	−0.3 (5)	C9—C10—C11—Fe1	59.6 (2)
C1—N1—C5—C4	−0.2 (5)	C10—C11—C12—C13	0.3 (3)
C1—N1—C5—N2	179.8 (3)	Fe1—C11—C12—C13	−58.9 (2)
C3—C4—C5—N1	0.2 (5)	C10—C11—C12—Fe1	59.2 (2)
C3—C4—C5—N2	−179.7 (3)	C11—C12—C13—C9	−0.9 (3)
N3—N2—C5—N1	−178.1 (2)	Fe1—C12—C13—C9	−60.1 (2)
C6—N2—C5—N1	4.1 (5)	C11—C12—C13—Fe1	59.1 (2)
N3—N2—C5—C4	1.8 (4)	C10—C9—C13—C12	1.1 (3)
C6—N2—C5—C4	−175.9 (3)	C8—C9—C13—C12	−178.9 (3)
N3—N2—C6—N4	177.0 (3)	Fe1—C9—C13—C12	59.8 (2)
C5—N2—C6—N4	−5.1 (5)	C10—C9—C13—Fe1	−58.7 (2)
N3—N2—C6—C7	0.0 (3)	C8—C9—C13—Fe1	121.3 (3)
C5—N2—C6—C7	177.9 (3)	C18—C14—C15—C16	0.6 (4)
N4—C6—C7—C8	−176.4 (3)	Fe1—C14—C15—C16	60.1 (2)
N2—C6—C7—C8	0.2 (3)	C18—C14—C15—Fe1	−59.5 (2)
N2—N3—C8—C7	0.4 (3)	C14—C15—C16—C17	−0.6 (4)
N2—N3—C8—C9	−179.8 (3)	Fe1—C15—C16—C17	58.9 (2)
C6—C7—C8—N3	−0.4 (4)	C14—C15—C16—Fe1	−59.5 (2)
C6—C7—C8—C9	179.8 (3)	C15—C16—C17—C18	0.5 (4)
N3—C8—C9—C10	−167.5 (3)	Fe1—C16—C17—C18	59.4 (2)
C7—C8—C9—C10	12.2 (5)	C15—C16—C17—Fe1	−58.9 (2)
N3—C8—C9—C13	12.5 (5)	C16—C17—C18—C14	−0.2 (4)
C7—C8—C9—C13	−167.7 (3)	Fe1—C17—C18—C14	59.6 (2)
N3—C8—C9—Fe1	102.0 (3)	C16—C17—C18—Fe1	−59.8 (2)
C7—C8—C9—Fe1	−78.2 (4)	C15—C14—C18—C17	−0.3 (4)
C13—C9—C10—C11	−0.9 (3)	Fe1—C14—C18—C17	−59.9 (2)
C8—C9—C10—C11	179.1 (3)	C15—C14—C18—Fe1	59.6 (2)

### Hydrogen-bond geometry (Å, º)

**Table:**

*D*—H···*A*	*D*—H	H···*A*	*D*···*A*	*D*—H···*A*
N4—H4*A*···N3^i^	0.86 (3)	2.44 (3)	3.210 (4)	150 (3)
N4—H4*B*···N1	0.88 (4)	2.05 (4)	2.749 (4)	136 (3)

Symmetry code: (i) *x*, −*y*+1/2, *z*−1/2.
